# Anomalous cerebral morphology of pregnant women with cleft fetuses

**DOI:** 10.3389/fnhum.2022.959710

**Published:** 2022-09-07

**Authors:** Zhen Li, Chunlin Li, Yuting Liang, Keyang Wang, Li Wang, Xu Zhang, Qingqing Wu

**Affiliations:** ^1^Department of Ultrasound, Beijing Obstetrics and Gynecology Hospital, Capital Medical University, Beijing Maternal and Child Health Care Hospital, Beijing, China; ^2^Beijing Advanced Innovation Center for Big Data-Based Precision Medicine, Capital Medical University, Beijing, China; ^3^School of Biomedical Engineering, Capital Medical University, Beijing, China; ^4^Beijing Key Laboratory of Fundamental Research on Biomechanics in Clinical Application, Capital Medical University, Beijing, China; ^5^Department of Radiology, Beijing Obstetrics and Gynecology Hospital, Capital Medical University, Beijing Maternal and Child Health Care Hospital, Beijing, China

**Keywords:** pregnant women, isolated clefts of the lip and/or palate (ICL/P), cortical thickness (CT), gray matter volume (GMV), magnetic resonance imaging (MRI), gyrification

## Abstract

**Objective:**

Pregnancy leads to long-lasting changes in brain structure for healthy women; however, little is known regarding alterations in the cortical features of pregnant women with malformed fetuses. Isolated clefts of the lip and/or palate (ICL/P) are the most common congenital anomaly in the craniofacial region, which is highly gene-associated. We speculated that pregnant women carrying fetuses with ICL/P may have associated risk genes and specific brain changes during pregnancy.

**Methods:**

In this study, we investigated T1-weighted brain magnetic resonance imaging data from 48 pregnant women: 24 women carrying fetuses with ICL/P (ICL/P group) and 24 women carrying normal fetuses (normal controls), then explored intergroup differences in gray matter volume (GMV), cortical thickness (CT) and cortical complexity (gyrification).

**Results:**

Compared with controls, the ICL/P group had decreased total intracranial volume (TIV) than normal controls; besides, they exhibited increased GMV in the left cuneus, decreased GMV in the right superior temporal gyrus; increased CT in the left precuneus and left superior parietal gyrus, decreased CT involving parsopercularis, fusiform, middle temporal in the left hemisphere and supramarginal, precentral gyrus (PreCG) in the right hemisphere; increased gyrification in the left insula and PreCG, the left middle temporal, and the right supratemporal gyrus.

**Conclusion:**

Pregnant women with ICL/P fetuses had brain morphology changes involving language, auditory, vision, and sensory cortex, which may be their special brain changes compared to normal pregnant women. This study may provide clues for the early detection of fetuses with ICL/P, and be vital for preconception and prenatal counseling with non-invasive methods.

## Introduction

Pregnancy leads to long-lasting structural and functional adaptations in the mother’s brain for non-human animals and humans, which are necessary for the onset, maintenance, and regulation of maternal behavior (Nelander et al., 2018; Feldman et al., 2019; Pawluski et al., 2022). Structural magnetic resonance imaging (MRI) on healthy primiparous women (first-time mothers) explored an increase of the pituitary gland (Gonzalez et al., 1988), transient reductions in overall brain size in pregnant women (Oatridge et al., 2002), and widespread reduced gray matter volume (GMV) covering the right temporal lobe, precuneus, prefrontal cortex compared with nulliparous, which was associated with decreased cognitive function (Hoekzema et al., 2017). Carmona et al. (2019) found a monthly rate of volumetric reductions of 0.09 mm^3^ for primiparous women, accompanied by decreases in cortical thickness (CT), surface area, local gyrification index, sulcal depth, and sulcal length, as well as increases in sulcal width. Furthermore, these motherhood-related brain structural changes predicted measures of postpartum maternal attachment (Hoekzema et al., 2020). There were also studies showing no significant changes in GMV, white matter volume (WMV), or brain volume for primiparous women during this period (Zheng et al., 2018).

At present, very little is known concerning maternal brain structural changes in pregnant women with malformed fetuses. This kind of research may provide important clues for preconception and prenatal counseling with non-invasive methods. Isolated clefts of the lip and/or palate (ICL/P) are the most common congenital anomaly in the craniofacial region, which is highly gene-associated. Numerous studies explored widespread brain structural changes in the ICL/P cohorts (Adamson et al., 2014; Conrad et al., 2015, 2021; Kuhlmann et al., 2021). We speculated that pregnant women carrying fetuses with ICL/P (pregnancies with ICL/P) may have associated risk genes and specific expression, for example, specific brain changes during pregnancy. Exploring these changes will add to the early detection of fetuses with ICL/P, and will be vital for preconception and prenatal counseling.

In our previous functional MRI (fMRI) study, pregnancies with ICL/P were shown to have altered functional connectivity and topological indices within neural networks of speech and language (Li et al., 2019). Brain morphological analysis is a stable and reliable method to investigate brain structures (Schmaal et al., 2017; Zhao et al., 2019). In this study, we analyzed their brain structural data and compare GMV, CT, and cortical complexity (gyrification) between pregnancies with ICL/P and pregnant women carrying healthy fetuses (normal controls). We speculated that pregnancies with ICL/P may have specific brain cortical indexes, which may reveal the structural basis for the altered functional indices shown in our previous fMRI study. Besides, these results may provide clues for the early detection of fetuses with ICL/P, and be vital for preconception and prenatal counseling. Furthermore, they might provide clues for interaction between fetuses and their mothers during pregnancy for future study.

## Subjects

This study recruited 24 pregnant women carrying fetuses with ICL/P (ICL/P group) and 24 pregnant women carrying healthy fetuses [normal controls (NC) group] in Beijing Gynecology and Obstetrics Hospital affiliated with Capital Medical University from January 2018 to December 2019. Age, educational levels, and gestation weeks (GW) of both groups were matched (Table 1). The enrollment criteria mainly included: singleton pregnancy, carrying ICL/P fetuses, or normal fetuses. All fetuses were without intrauterine growth restriction or chromosome abnormality. The key exclusion criteria for mothers were brain structural abnormalities, neurological or psychiatric disorders, complications of pregnancy, and MRI contraindications. The research protocol was approved by the Medical Research Ethics Committee of Beijing Gynecology and Obstetrics Hospital. The methods were carried out in accordance with the Declaration of Helsinki. All participants provided written informed consent after being informed of the study details.

**TABLE 1:** Demographic data of the isolated clefts of the lip and/or palate (ICL/P) and normal controls (NC).

	ICL/P (24)	NC (24)	*p*
Age (year)	30.88 ± 4.18	31.71 ± 3.33	0.449^b^
Education (year)	15.0 (11–15.0)	15.0 (11.0–18.0)	0.095^a^
Gestation weeks (week)	24.96 ± 2.02	25.88 ± 2.16	0.133^b^
TIV (cm^3^)	1386.50 ± 66.49	1441.76 ± 107.24	0.037^b^

ICL/P, isolated clefts of the lip and/or palate; NC, normal controls; TIV, total intracranial volume. Normally data is presented as mean ± SD. Non-normally data is presented as median (Interquartile range, IQR). *p* < 0.05 indicates statistically significant.

^a^The Mann–Whitney U-test for non-normally distributed data between two groups.

^b^The two-sample t-test for normally distributed data between the two groups.

### Magnetic resonance imaging scan protocol

Brain MRI scans were performed using a 3.0-T MR scanner (Discovery MR750, GE, Milwaukee, WI, United States), 32-channel head coil. Participants were placed on their backs so that they remained relaxed during the scan. A cushion was used to limit head movement. The T1-weighted brain structure scan parameters were as follows: repetition time = 8.2 ms; echo time = 3.2 ms; flip angle = 12; acquisition matrix = 256 × 256; voxel size = 1 mm × 1 mm × 1 mm; 164 contiguous axial slices.

### Structure magnetic resonance imaging preprocessing

Brain MRI data were processed with the Computational Anatomy Toolbox 12 (CAT12^1^) and Statistical Parametric Mapping12 (SPM12^2^). Images were processed with the following steps: converted to Neuroimaging Informatics Technology Initiative (NIFTI) files, corrected for bias–field inhomogeneities, spatially normalized (using the Diffeomorphic Anatomical Registration using Exponentiated Lie algebra (DARTEL) algorithm (Ashburner, 2007)), segmented [into gray matter (GM), white matter (WM), and cerebrospinal fluid (CSF)] (Ashburner and Friston, 2005). Then GM maps were modulated and smoothed with a Gaussian kernel of 8 mm (full-width at half maximum). CT was computed with topological correction (based on spherical harmonics) (Yotter et al., 2011) and the established novel algorithm (for extracting the cortical surface) (Dahnke et al., 2013). The local curvature-based gyrification index was calculated based on absolute mean curvature (AMC) (Luders et al., 2006). Central cortical surfaces were created for both hemispheres separately. Surface-based images were resampled and smoothed with a Gaussian kernel of 20 mm (full-width at half maximum).

### Statistical analysis

Demographics data were compared using SPSS 22.0 software and *p* < 0.05 was considered statistically significant. Normally distributed data were expressed as mean ± SD, and tested by a two-sample *t*-test; non-normal distributed data were expressed as median (interquartile range, IQR), and tested by Mann–Whitney U-test non-parametric test.

The GMV maps of the two groups were compared using Matlab2013b and SPM12 with a two-sample *t*-test. Intergroup differences in CT and gyrification were performed using CAT12 and SPM12 with a two-sample *t*-test, across each hemisphere. Total intracranial volume (TIV, for GMV comparison), age and GW, and educational years were added as covariates. Statistical significance was defined as *p* < 0.001, uncorrected with a minimum cluster extent of 10 voxels (for GMV); *p* < 0.001, uncorrected or *p* < 0.05, peak-level Family Wise Error (FWE)-corrected with a minimum cluster extent of 20 vertices (for CT and gyrification).

## Results

### Demographic data

There were no statistical differences in age, GW, and educational background between the ICL/P and NC groups (Table 1).

### Intergroup gray matter volume difference between the isolated clefts of the lip and/or palate and normal controls groups

Compared with controls, the ICL/P group had decreased TIV than the NC group (Table 1). Besides, they exhibited increased GMV in the left cuneus and decreased GMV in the right superior temporal gyrus (*p* < 0.001, uncorrected, Supplementary Figure 1 and Supplementary Table 1).

### Intergroup cortical thickness difference between the isolated clefts of the lip and/or palate and normal controls groups

**FIGURE 1:**
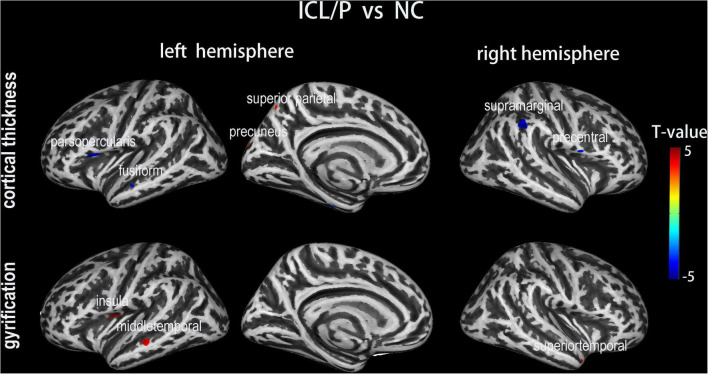
Altered cortical thickness (CT) and gyrification between the isolated clefts of the lip and/or palate (ICL/P) group and normal controls (NC). A two-sample *t*-test was used; statistical significance was *p* < 0.001, uncorrected. Color bars indicate T-values. The 3D inflated brain maps show the spatial location of intergroup different regions, shown on lateral or medial views.

**FIGURE 2:**
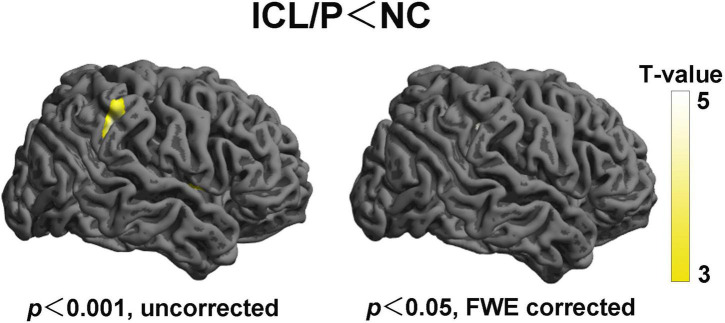
Altered cortical thickness (CT) of the right hemisphere between the isolated clefts of the lip and/or palate (ICL/P) group and normal controls (NC). Statistical significance was *p* < 0.001, uncorrected (left) and *p* < 0.05, peak-level FWE-corrected (right). Color bars indicate T-values. The 3D brain maps [supplied by SPM12 (Statistical Parametric Mapping)] show the spatial location of intergroup different regions, shown on lateral views.

Two-sample *t*-test showed that the ICL/P group exhibited increased CT in the left precuneus and left superior parietal gyrus, decreased CT involving parsopercularis, fusiform, middle temporal in the left hemisphere and the right precentral gyrus (PreCG) (*p* < 0.001, uncorrected, Figure 1 and Table 2). Furthermore, they had decreased CT in the right supramarginal gyrus (SMG) (*p* < 0.05, peak-level FWE-corrected, Figure 2 and Table 2). CT of the intergroup differential regions of both groups was displayed in Supplementary Figure 2.

**TABLE 2:** Intergroup cortical thickness (CT) difference between the isolated clefts of the lip and/or palate (ICL/P) and normal controls (NC).

T-value	Cluster size		Overlap of atlas region
ICL/P > NC			
3.6	50	100%	Precuneus (L)
3.4	36	94%	Superior parietal (L)
		6%	Cuneus (L)
ICL/P < NC			
4.1	273	100%	Parsopercularis (L)
3.5	107	91%	Fusiform (L)
		9%	Parahippocampal (L)
3.7	43	100%	Middle temporal (L)
4.6*	440	100%	Supramarginal (R)
3.6	92	100%	Precentral (R)

ICL/P, isolated clefts of the lip and/or palate; NC, normal controls. A two-sample t-test was performed between ICL/P and NC. Results were thresholded at *p* < 0.001, uncorrected. Results passed *p* < 0.05, peak-level FWE-corrections were marked with asterisk. (L)/(R) means left/right hemisphere. Atlas labeling was performed according to the Desikan-Killiany atlas (Desikan et al., 2006).

### Intergroup gyrification difference between the isolated clefts of the lip and/or palate and normal controls groups

The ICL/P group exhibited increased gyrification in the insula, middle temporal in the left hemisphere, and superiotemporal gyrus in the right hemisphere (*p* < 0.001, uncorrected, Figure 1 and Table 3). Gyrification of the intergroup differential regions of both groups was displayed in Supplementary Figure 2.

**TABLE 3:** Intergroup gyrification difference between the isolated clefts of the lip and/or palate (ICL/P) and normal controls (NC).

T-value	Cluster size		Overlap of atlas region
ICL/P > NC			
3.5	201	67%	Insula (L)
		33%	Precentral (L)
3.5	168	100%	Middle temporal (L)
3.5	58	100%	Superiotemporal (R)

ICL/P, isolated clefts of the lip and/or palate; NC, normal controls. A two-sample t-test was performed between ICL/P and NC. Results were thresholded at *p* < 0.001, uncorrected. (L)/(R) means left/right hemisphere. Atlas labeling was performed according to the Desikan-Killiany atlas (Desikan et al., 2006).

## Discussion

At present, very little is known concerning brain structural changes in pregnant women with malformed fetuses. In this study, we analyzed brain structural data and compare GMV, CT, and gyrification of pregnant women carrying fetuses with ICL/P (pregnancies with ICL/P) with normal controls. We found that pregnancies with ICL/P have brain morphology changes involving language, auditory, vision, and sensory cortex. These results might reveal the structural basis for the altered functional indices shown in our previous fMRI study; besides, these results may provide clues for the early detection of fetuses with ICL/P, and may be vital for preconception and prenatal counseling with non-invasive methods.

Compared with controls, the ICL/P group had decreased CT in regions involved in the language neural circuit (the left parsopercularis and the right SMG). The language neural circuit includes the Broca area, Wernicke’s area, SMG, angular gyrus, and the main long associated fibers connecting different language centers (Hage and Nieder, 2016; Hagoort, 2017). Broca’s area classically comprises cytoarchitectonic areas 44 (parsopercularis) and 45 (pars triangularis) in the left hemispheres, complemented by some authors by area 47 (pars orbitalis) (Ardila et al., 2017). Areas 44 and 45 on the left side of the brain are instrumental for the production, or articulation, of speech and language (Friederici and Gierhan, 2013). The parsopercularis is connected with the oral cavity and tongue movement area, it participates in language production and speech processing. The triangle part (BA45) participates in semantic processing (Turken and Dronkers, 2011). In addition, the ICL/P group had decreased CT in the right SMG (*p* < 0.05, peak-level FWE-corrected). SMG was shown to participate in auditory memory processing and speech decision-making (Mirman et al., 2015). Our previous work showed that for adults with cleft of lip and palate (CLP) after surgery, during articulation rehabilitation (focusing on improving articulation clarity), the junction part of the right postcentral gyrus and right SMG was involved, which indicated right SMG was a key region for articulation improving (Li et al., 2020).

Besides, we found decreased CT in the right PreCG and increased gyrification in the left PreCG in the ICL/P group. Our previous fMRI study showed that the functional connection between PreCG and amygdala in both hemispheres declined in pregnant women with ICL/P fetuses compared with controls (Li et al., 2019). Decreased CT and increased gyrification of PreCG in both hemispheres may have explored its structural basis.

In summary, pregnant women with ICL/P fetuses (ICL/P group) have brain morphology changes involving language, auditory, vision (fusiform gyrus), and sensory cortex (right PreCG), which may be brain changes in the ICL/P group compared to normal pregnant women. This may lead to differences in their language or auditory or cognition, just as findings in the ICL/P cohorts (Conrad et al., 2015, 2021; Kuhlmann et al., 2021), while at present the cognitive and language status of parents of the ICL/P cohorts stay unknown. This may be a direction for future research. Meanwhile, we speculated that the altered cerebral morphology of pregnant women with cleft fetuses may be caused by carrying CL/P risk genes. This study provided an important supplement to the limited research on maternal brain structure for the ICL/P cohorts. Brain morphology changes of the ICL/P group may give support to the theory that abnormal migration of cells caused during facial development occurs concurrently with abnormal migration of neuronal cells. Large samples and genetic studies will be needed to supply more confirmation.

The present study also had several limitations. Firstly, the sample size of this study was small, besides, most of the results were at the uncorrected statistic level and they did not survive under multiple comparisons correction. As a result, they cannot be considered reliable and need to be carefully interpreted. Secondly, this study is a cross-sectional study, without the comparison of structural data before and after pregnancy. The results of the longitudinal study will be more convincing. This will be the future direction.

## Data availability statement

The raw data supporting the conclusions of this article will be made available by the authors, without undue reservation.

## Ethics statement

The studies involving human participants were reviewed and approved by the Medical Research Ethics Committee of Beijing Gynecology and Obstetrics Hospital (No. 2017-KY-085-02). The patients/participants provided their written informed consent to participate in this study.

## Author contributions

ZL contributed to study conception, design, data analysis, and interpretation, statistical analyses, and manuscript writing. CL contributed to conception, design, interpretation, and manuscript writing. YL, KW, and LW contributed to the design, participant recruitment, and data acquisition and interpretation. XZ and QW contributed to the conception, design, data acquisition and interpretation, and manuscript review and modification. All authors approved the final manuscript.

## Footnotes

1
https://neuro-jena.github.io/cat//index.html#DOWNLOAD

2
https://www.fil.ion.ucl.ac.uk/spm/software/spm12/

## Supplementary material

Supplementary Figure 1 Altered gray matter volume (GMV) between the isolated clefts of the lip and/or palate (ICL/P) group and normal controls (NC). A two-sample *t*-test was used; statistical significance was *p* < 0.001, uncorrected. Color bars indicate T-values. The brain maps show the spatial location of intergroup different regions, shown on a cross or coronal views.

Supplementary Figure 2 Cortical thickness (CT) and gyrification of the intergroup differential regions of the isolated clefts of the lip and/or palate (ICL/P) group and normal controls (NC). The statistical level of the intergroup differential regions was *p* < 0.001, uncorrected. (L)/(R) means left/right hemisphere.
