# Supplementary material for: Anomalous cerebral morphology of pregnant women with cleft fetuses
Source: Front Hum Neurosci. 2022 Sep 7;16:959710. doi: 10.3389/fnhum.2022.959710 (PMC9491019; doi:10.3389/fnhum.2022.959710)
Supplement: Supplementary file 1 [file Table_1.DOCX]

| Brain regions | AAL Area | Peak MNI coordinates | | | T-value | Cluster size |
| --- | --- | --- | --- | --- | --- | --- |
|  |  | x | y | z |  |  |
| ICL/P＞NC |  |  |  |  |  |  |
| Left Cuneus | Cuneus_L | -11 | -87 | 24 | 3.633 | 13 |
| ICL/P＜NC |  |  |  |  |  |  |
| Superior Temporal Gyrus | Temporal_Sup_R | 74 | -12 | 9 | -3.833 | 15 |

Table S1 Intergroup GMV difference between the ICL/P and NC group

A two-sample t-test was performed between the ICL/P and NC group. Results were thresholded at *P*＜0.001, uncorrected. Atlas labelling was performed according to the AAL atlas[1].

[1] Tzourio-Mazoyer N, Landeau B, Papathanassiou D, Crivello F, Etard O, Delcroix N, et al. Automated anatomical labelling of activations in spm using a macroscopic anatomical parcellation of the MNI MRI single subject brain. Neuroimage 2002; 15: 273-289.
