# Supplementary figures and images for: Anomalous cerebral morphology of pregnant women with cleft fetuses
Source: Front Hum Neurosci. 2022 Sep 7;16:959710. doi: 10.3389/fnhum.2022.959710 (PMC9491019; doi:10.3389/fnhum.2022.959710)

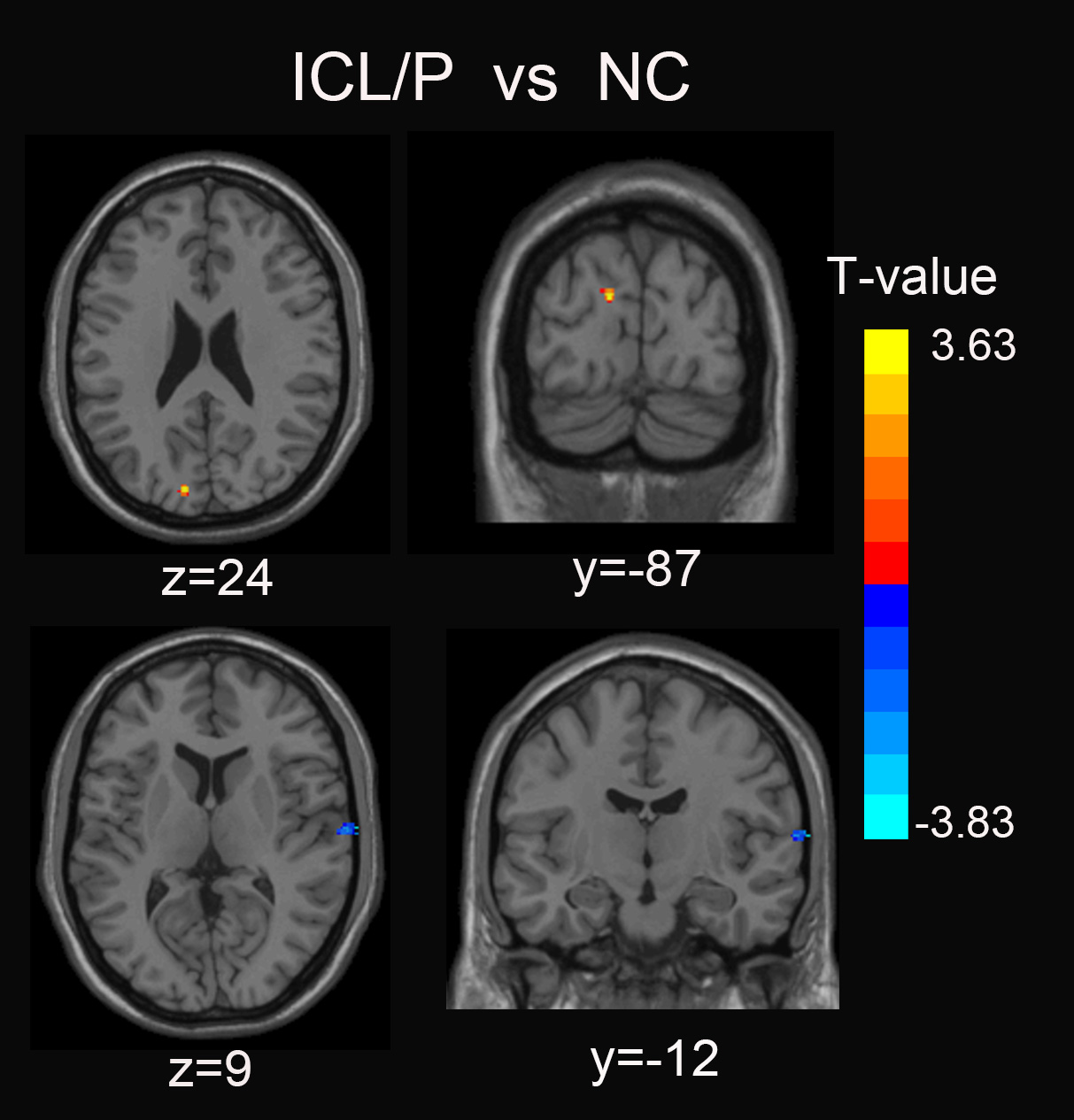

Supplement: Supplementary Figure 1 — Altered gray matter volume (GMV) between the isolated clefts of the lip and/or palate (ICL/P) group and normal controls (NC). A two-sample t-test was used; statistical significance was p < 0.001, uncorrected. Color bars indicate T-values. The brain maps show the spatial location of intergroup different regions, shown on a cross or coronal views. [file Image_1.JPEG]

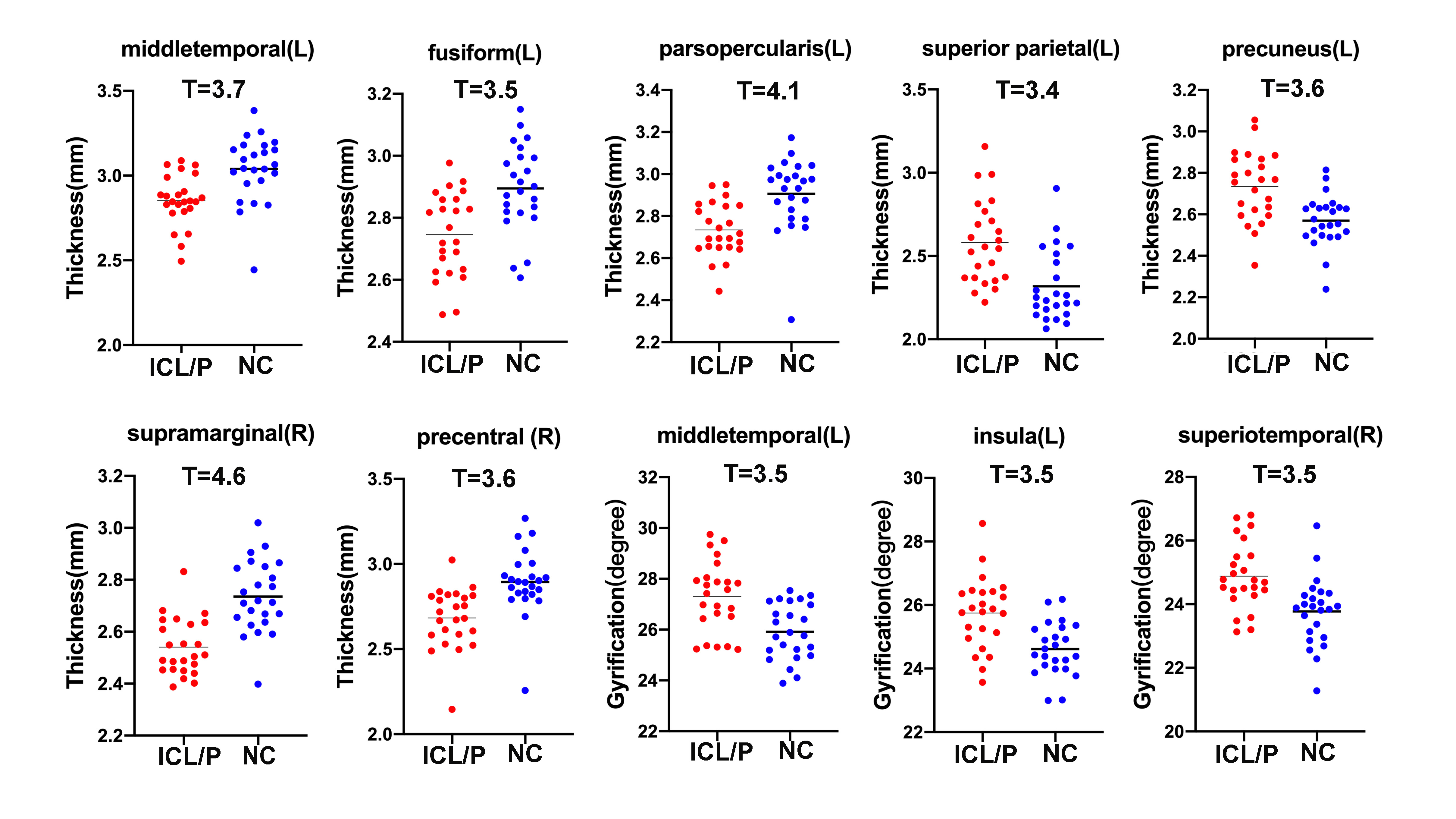

Supplement: Supplementary Figure 2 — Cortical thickness (CT) and gyrification of the intergroup differential regions of the isolated clefts of the lip and/or palate (ICL/P) group and normal controls (NC). The statistical level of the intergroup differential regions was p < 0.001, uncorrected. (L)/(R) means left/right hemisphere. [file Image_2.JPEG]
